# Effect of a locally adapted genome on environmentally induced epigenetic variation

**DOI:** 10.1093/eep/dvy025

**Published:** 2018-11-26

**Authors:** France Beauregard, Bernard Angers

**Affiliations:** Departement of Biological Sciences, Université de Montréal, Montréal, Canada

**Keywords:** epigenetics, genetic variation, genome exchange, kleptogenesis, unisexuals, phenotypic plasticity, *Ambystoma laterale–jeffersonianum*

## Abstract

Both genetic variation and environmentally induced epigenetic changes allow organisms to persist through the heterogeneity of their habitats. Selection on genetic variation can promote local adaptation of populations. However, in absence of genetic variation, clonal organisms mostly rely on epigenetics to respond to environmental heterogeneity. We used the potential of unisexual organisms in incorporating their host genome, to empirically assess whether the presence of a locally adapted genome affects environmentally induced epigenetic changes in clonal organisms. We addressed this problematic by using unisexual lineages of the kleptogen vertebrate *Ambystoma laterale–jeffersonianum* complex that can optionally incorporate genetic material from locally adapted sexual hosts through genomic exchanges. More specifically, we compared environmentally induced epigenetic changes between lineages strictly reproducing clonally vs. those incorporating a locally adapted genome. The results revealed that both lineage and sample site components, as well as their interaction, affected epigenetic variation. When lineages were analysed separately, differences among sample sites were only detected in lineages impervious to genomic exchanges. Sample sites had no significant effect on the epigenetic variation of lineages that performed genomic exchanges. These results suggest that environmentally induced epigenetic variation among sites depends more on the lack of locally adapted alleles than on the level of genetic variation.

## Introduction

Organisms have developed a wide range of adaptive strategies to face environmental fluctuations [1]. Most have in common the need to produce phenotypic variation, and the main consistent sources of such diversity are genetic and epigenetic variation. Random recombination of DNA mutations through sex maximizes the number of phenotypic alternatives through generations, representing the heritable source of phenotypic variation.

Epigenetics consists of enzyme-mediated chemical modifications of histones, DNA and RNA. These changes modify the properties of these molecules while the amino/nucleic acids sequence remains unchanged [2–4]. These changes may therefore affect phenotype through multiple pathways, including variation in gene expression. Epigenetic modifications can occur stochastically, like mutations, but at higher rates [5–7]. They can also be induced by environmental changes [2] and thus constitute the mechanism behind phenotypic plasticity [3]. Both sources of epigenetic variations have been assigned to distinct strategies according to the rate of the environmental changes [8–10]. Most epigenetic changes are erased and re-established at each generation [11], allowing progeny to face environmental conditions potentially different from those of their parents.

The relative importance of epigenetic vs. genetic variation is comparable to the question of the relationship between phenotypic plasticity and genetic diversity which has been strongly debated in the literature. Some authors have argued that phenotypic plasticity is negatively correlated with genetic diversity because phenotypic plasticity evolves to display phenotypes acclimated to different environments without resorting to genetic variation [12–15]. Others have proposed the opposite since phenotypic plasticity would diminish the influence of natural selection on the genotype, leading to an increase in genetic variation [16]. However, a third group has found no relationship between genetic variation and phenotypic plasticity, arguing that those characteristics evolve independently [17–19].

To empirically assess the relative importance of genetic and epigenetic variation, one could compare genetically similar individuals with or without the presence of a locally adapted genome. This allows isolation of the genetic effects from environmentally induced epigenetic variation. A locally adapted genome results of a long-term selection on genetic variation, providing to a population a higher fitness in their native environment than in other environments.

Such particular system can naturally occur in unisexual vertebrates. These organisms reproduce clonally while they need sperm of a host species to trigger egg’s development. However, they can occasionally acquire a haplome of the sexual host via paternal leakage; when the sperm’s genome is incorporated to the egg’s pronucleus [20]. If the host population is locally adapted, the genetic material transferred to clones is expected to increase their fitness.

Kleptogenesis is an example of a mechanism used by unisexual organisms to occasionally incorporate genetic material of a closely related species [21]. Kleptogenetic females may produce reduced and unreduced eggs, to which the male genome may or may not be added. The production of a reduced egg paired with male genome inclusion results in a full set of chromosomes (haplome) of the mother replaced by the haplome of the sperm. This process is called genome replacement [21, 22]. Alternatively, an unreduced egg paired with the rejection of the male genome is equivalent to gynogenesis and leads to clonal reproduction. The two other combinations of events produce offspring with ploidy reduction (reduced egg and male genome rejection) or ploidy elevation (unreduced egg and male genome inclusion). Ploidy reduction followed by ploidy elevation—and *vice versa*—leads to the same outcome as genome replacement, but over several reproduction events. Genome exchange is the term used to designate the resulting haplome permutation, regardless of whether it occurred in one reproduction event or over many generations [23].

The objective of this study was to assess whether the presence of a locally adapted genome affects environmentally induced epigenetic variation in a kleptogen vertebrate. The unisexual of the *Ambystoma laterale–jeffersonianum* complex was used as a biological model because genome exchange has been inferred in multiple locations [21, 24–27]. Moreover, in the northern part of its distribution, lineages performing genome exchanges (GE+) coexist with strictly clonal lineages (GE−) [27, 28]. The presence of both genetically diverse and clonal populations provides a suitable system to assess the importance of a locally adapted genome for epigenetic variation.

Individuals of a given GE− lineage are expected to display distinct epigenetic profiles in response to the environmental conditions of different sampling sites. However, since the locally adapted haplome incorporated through genome exchange might be partly responsible for the phenotype of a GE+ lineage, we predict a lower contribution of environmentally induced epigenetic variation among sites.

## Methods

### Sampling, DNA Extraction and Genotype Determination

**Figure 1:**
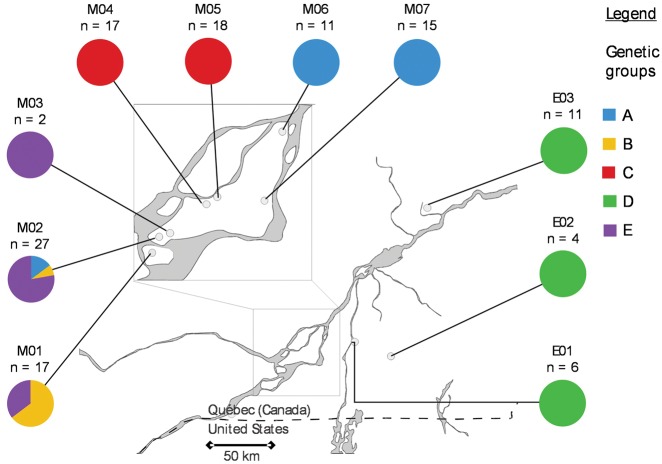
geographic distribution of the unisexual lineages sampled for epigenetic analyses. The region in close-up represents the Montreal region. Colors refer to the different lineages previously inferred [28]

Individuals were collected in a study previously conducted by Beauregard and Angers [27]. Sampling was conducted over two consecutive years during the reproduction period at 10 sites located in southern Quebec (Fig. 1). Minnow traps were placed in ponds overnight, and salamanders were collected in the morning. Individuals captured were anesthetized, and a tail tip was collected and preserved in ethanol. DNA extraction was performed according to the phenol–chloroform purification and ethanol precipitation method of Sambrook *et al.* [29]. The unisexual *Ambystoma laterale–jeffersonianum* harbored a hybrid genome including at least one haplome from *A. laterale* and one from *A. jeffersonianum.* An additional haplome of *A. laterale*, the unique host species in this region, characterized most of the individuals captured. Lineages were previously discriminated using microsatellite loci [27]. Strictly clonal lineages (GE−) were characterized by the absence of genetic variation among individuals while those performing genome exchanges (GE+) were genetically diversified and shared alleles with the local host species [27].

A representative subsample of 125 unisexuals was selected for epigenetic analyses to maximizing comparison of lineages among sites (Fig. 1, Supplementary Table S1). Most sites were represented by a single (M03, M06, M07, E01–03) or largely dominated by one (M04, M05) lineage (Fig. 1). Triploids (LLJ) represented 78.9% of the subsample, while diploids (LJ) and tetraploids (LLLJ) represented respectively 15.6% and 5.5% (Fig. 1, Supplementary Table S1). Two sites were only represented by diploid populations (E01 and E02).

### Environmental Characterization

To determine whether environmental conditions among sampling sites of a given lineage were different, we performed an environmental characterization of the aquatic and terrestrial environments of the sites. First, environmental conditions were measured during the first sampling season. All sites (except M01 and M03, sampled during the second year) were visited four times during the larval development period from May to August 2014 (Supplementary Table S2). We measured aquatic variables in the reproductive ponds: pH; oxygen concentration; conductivity; oxydo-reduction potential; variation of the water level; nature of the substratum; presence of water connection, herbaceous plants, and trees; and percentage of tree cover above the pond. We also measured terrestrial variables of the adjacent forest in two quadrates of 25 × 25 m: soil pH, drainage score, percentage of coniferous trees, and coverage level of the shrub and tree layers.

We completed the characterization with other sources of public data. The forest maps (Ministry of Forests, Wildlife, and Parks of Quebec) provided data on age class, density, height, and inclination indices, as well as main and second tree essence near the site. Tree essences were related to a sunlight, soil acidity, and humidity indices. Detailed mapping of wetlands by *Ducks Unlimited Canada* was also used to further characterize the reproductive pond: type of wetland (swamp, marsh, or pond), superficies, and permanence. Finally, the soils characterization databank (*Institut de recherche et de développement en agroenvironnement*) was used to extract data about the tilt profile; soil drainage; granulometry; pH; dominance of clay, loam, silt, or sand; soil alkalinity, and humidity indices; and the percentage of coarse fragments, very fine sand, sand, silt, clay, and organic carbon. Data were only available for sites M01-05, M07, and E03.

All data-sets included various types of data. Quantitative data were not transformed otherwise. Semi-quantitative data in the form of descriptor indices were associated with a number from 0 to the higher descriptor. Semi-quantitative data in the form of different numerical classes were converted to the median of each class. Unordered qualitative data were split into as many columns as there were categories and converted to binary data [30].

The four different databanks were analysed separately, since they do not all include data of the same sites (Supplementary material S6–S9). A global comparison of the environmental conditions among the different sampling sites was performed using Principal Component Analysis (PCA) with the package vegan [31]. The relative Euclidian distance of environmental variables between sites was computed according to their scaled environmental variables.

### Epigenetic Analyses

The Methylation Sensitive Amplified Polymorphism (MSAP) method was chosen because it allows the coverage of a large-spectrum portrait of the epigenetics of a large number of individuals. A total of 125 individuals were selected to conduct MSAP analysis according to a protocol modified from Xiong *et al.* [32]. The MspI and HpaII enzyme cuts DNA differently according to the methylation state of the internal cytosine, so the resulting band pattern is representative of the genetic and the epigenetic profiles, respectively. In addition to the frequent cutter, a rare cutter, KpnI, was used. Preamplification targeted a selection of two nucleotides and involved 5′-ACGATGAGTCCTGAGCGGCC for MspI-CC extremities and 5′-GTAGACTGCGTACCGTACCGC for KpnI-GC extremities. Selective amplification required the following primers: 5′-GATGAGTCCTGAGCGGCCGC for MspI-CCGC extremities (the only used combination because of the significantly clearer results) and 5′ GACTGCGTACCGTACCGCNN for KpnI-GCNN extremities (variable combinations: GCTA, GCTT, GCAA, GCAG). To ensure the reproducibility of the results, at least two replications of each selective amplification were performed on all individuals and only loci with an unambiguous and consistent signal for both replications were kept for analysis.

The epigenetic matrix was obtained by removing all the loci in the presence–absence HpaII matrix that matched loci in the MspI matrix to avoid variation correlated with genetics (Supplementary : Sheet S1). The simple-matching coefficient was used to calculate the distance matrix from the presence–absence AFLP matrix to account for both double presences and double absences. To visualize the repartition of the individuals according to their epigenetic patterns, the inter-individual distance was represented via a principal coordinates analysis (PCoA) using the function *cmdscale* in R.

Variance partitioning was computed for all individuals from the presence–absence epigenetic matrix with the function varpart of the package vegan. Sample sites, ploidy level, and genetics were considered as constraining matrices (Supplementary Sheets S2–S4). The descriptive genetic matrix was a compilation of presence–absence of the highly divergent microsatellites’ alleles (see [27]) and the MspI genetic matrix. Environment matrix refers to a sampling site as qualitative data. Direct correlation between environmental variables and epigenetics was not considered in the absence of predictions between the scored epigenetic loci and the environmental variables we recorded.

Total and partial environmental effect for each lineage was also tested with the functions rda and Rsquare Adj of the package vegan. For total environmental effect, only site of sampling was considered as a constraining matrix. For partial environmental effect, site of sampling, ploidy level, and intra-lineage genetics were all considered as constraining matrices. The *P*-value of partial and total environmental effect on epigenetic patterns was obtained with the function anova.cca of the package vegan. Total and partial environmental effect for all lineages with genome exchange (B, C, and E) and all exclusively clonal lineages (A and D-2n) was computed using the same method.

## Results

### Environmental Characterization

**Figure 2:**
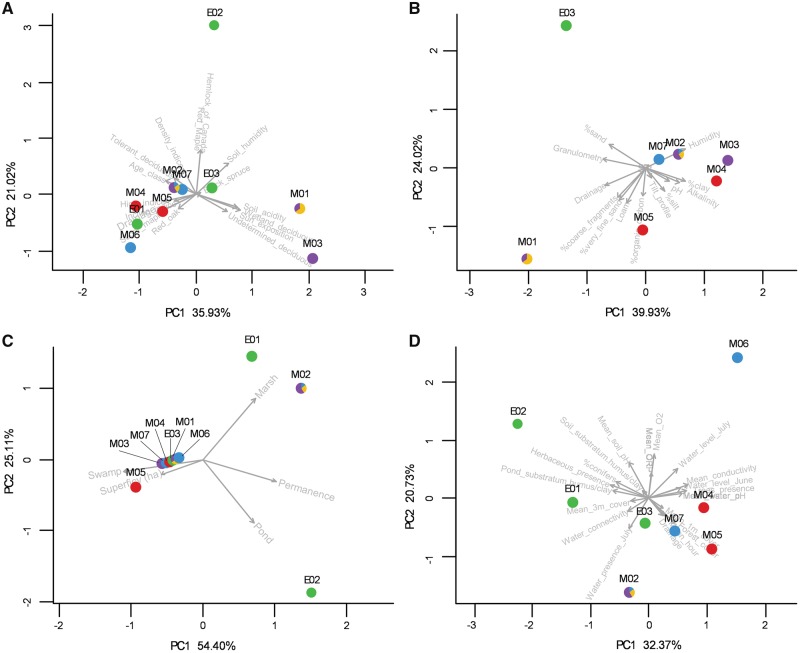
distribution of the sampling sites according to environmental conditions. Results of the principal component analyses according to (**A**) variables from the forest maps, (**B**) soils characterization (sites M06, E01, and E02 are absent), (**C**) the interactive maps of detailed mapping of wetlands, and (**D**) characterization of the sites’ conditions performed in this study (sites M01 and M03 are absent). Colors represent the presence of lineages at a given site. Arrows represent the explanatory vectors

The sites clustered differently according to the different datasets used for environmental characterization (Fig. 2). Sites that included individuals from the same lineage could be similar for a given condition but different for other conditions. Only the sites where lineage D was found (E01, E02, and E03) were strongly different from each other regardless of the data-set used for the analyses.

All sites could be assigned to one of the five categories of environmental conditions. Sites that included individuals from the same lineage belonged to different categories. M02 and E02 are semi-permanent ponds created by digging and connected to a drainage ditch. E01 and M06 are temporary ponds easily drained in the top of a small mountain. M07 is a large marshy stream with some fish. M01 and M04 are larger, better defined, and deeper arborescent swamps, while M03, M05, and E03 are a grouping of a few smaller arborescent swamps.

### Epigenetic Variation

**Figure 3:**
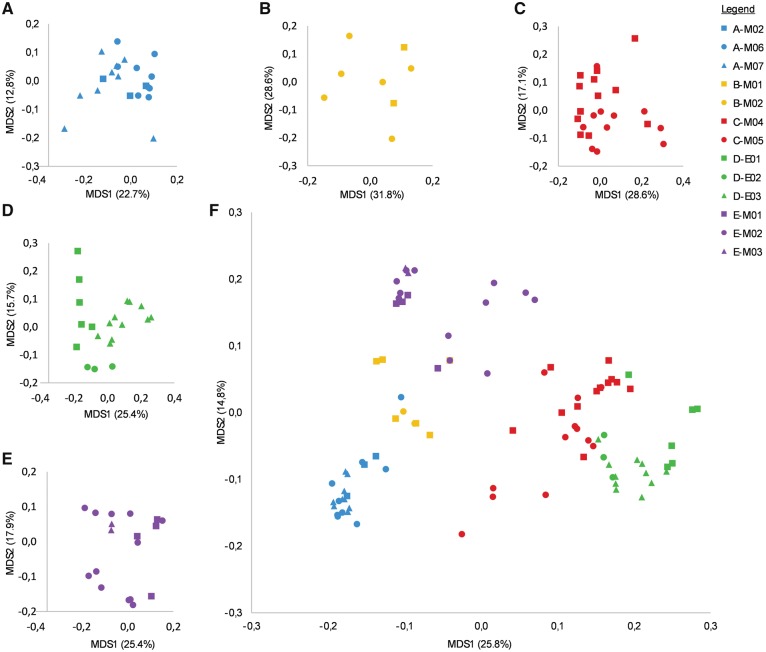
distribution of unisexual individuals according to their epigenetic patterns. Results of the principal coordinates analyses (**A–E**) for lineages A–E, and (**F**) for all individuals. Color represents each lineage, and the form-color combination represents the sampling site origin. Percentage of representativity of axes 1 and 2 are indicated aside

A total of 52 loci of the methylation pattern were kept for analysis, of which 37 were variable and 27 were informative (Table 1). Principal coordinates analysis performed on epigenetic profiles revealed that individuals of a given lineage are different according to sampling sites (Fig. 3A–E). When analysed altogether, most of the individuals primarily clustered according to their lineage (Fig. 3F).

**Table 1:** epigenetic variation among lineages

Lineage	*n*	Variable loci	(%)	Informative loci	(%)
A	30	**21**	**40.4**	**16**	**30.8**
B	13	7	13.5	6	11.5
C	33	16	30.8	12	23.1
D	21	**21**	**40.4**	**20**	**38.5**
E	28	**20**	**38.5**	9	17.3
**Mean**		17	33	13	24
**Total**	125	37	71.2	27	51.9

Sample size (*n*) as well as number (percentage) of variable loci per lineage (at least one individual is different) and informative loci (at least two individuals display the same difference) are provided. The values in bold characters are above the mean of the comparison between the lineages.

**Figure 4:**
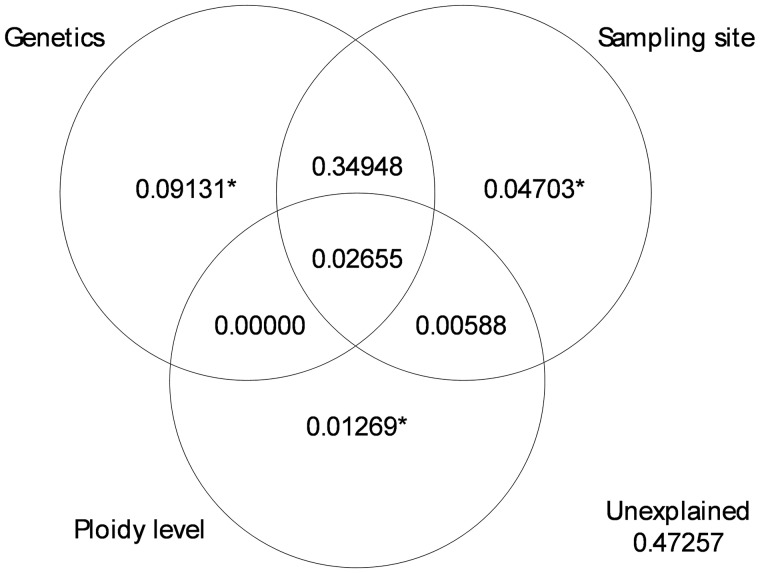
partition of the epigenetic variance according to genetics, sampling site, and ploidy level data. The values represent the adjusted *R*-squared. *Asterisk refers to *P*-value < 0.001. The shared effects and the unexplained variation cannot be tested for significance

Variance partitioning on all individuals supports these trends (Fig. 4). The total effects of genetics (46.2%) and environment (42.9%) are both strong and of similar extent, while ploidy level has a very small effect (3.96%). However, a large part of these effects is shared between genetics and sampling sites (34.9%); the pure effect of each component is therefore considerably smaller but remains significant. The genetic effect is strong when all individuals are analysed together due to the differentiation of lineages. The genetic effect is not significant within the lineages, except for lineage C (Supplementary Table S3).

**Figure 5:**
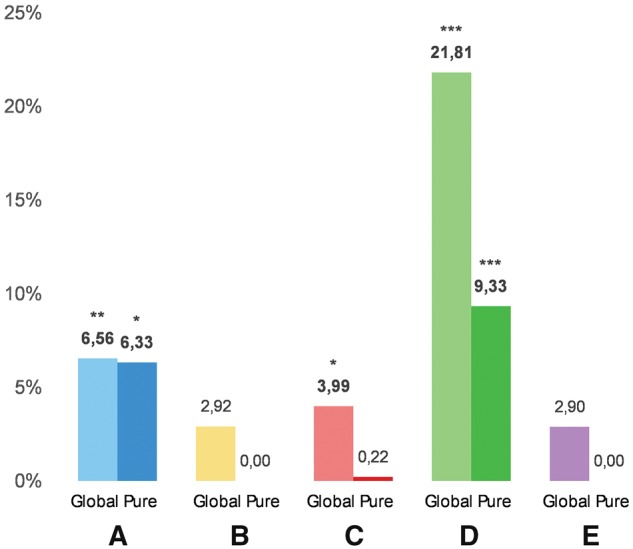
effect of the sampling site on the epigenetic variation per lineage. Epigenetic variation explained by sample site (total effect: light bars) or controlled for shared effect between sample sites vs. genetics and ploidy level (pure effect: darker bars). The letters (**A**–**E**) refer to the lineage. The values above the bars are the percentage of variation explained, derived from the adjusted *R*-squared. Statistical significance (*P*-value): * < 0.05, ** < 0.01, *** < 0.001

When lineages are analysed separately, sampling sites have a significant effect on three lineages (Fig. 5; A: 6.6%, C: 4.0%, D: 21.8%). However, when the effects of genetics and ploidy level are controlled, sampling sites have a significant effect on only two lineages (A: 6.3%, D: 9.3%).

When genetic variation and ploidy levels are taken into account, sampling sites are only significant for the exclusively clonal lineages analysed together (A and D-2n; *R*^2^_adj_ = 0.06, *P*-value < 0.001), whereas they are not significant for the lineages with genome exchange analysed together (B, C, and E; *R*^2^_adj_ = 0.004, *P*-value = 0.247).

## Discussion

The objective of this study was to assess the effects of locally adapted genetic variation on environmentally induced epigenetic changes in the kleptogen *Ambystoma laterale–jeffersonianum*. We predicted that genetically identical individuals from different sites would display distinct patterns of environmentally induced epigenetic variation in response to different environmental conditions. In contrast, lineages that had incorporated a locally adapted genome from a local sexual host could benefit from a more well-suited phenotype and rely less on epigenetic variation.

As expected, the effect of the sample sites, as a proxy for the environments, differed among the five lineages. The three lineages that performed genome exchange (GE+: lineages B, C, and E) did not display significant epigenetic differences among sites once the effects of genetics and ploidy were removed. On the contrary, sample sites had a significant effect only on the clonal lineages (GE−: lineages A and D-2n).

### Environmental Conditions

Difference in environmental conditions among sampling sites is a crucial factor to consider when assessing environmentally induced epigenetic variation [33–37, and others reviewed in 38]. However, in this study, the environmental conditions of the sample sites were not directly considered in the analyses due to the difficulty in characterizing determinant factors during the life cycle of these salamanders. Several ecological and physicochemical factors of the natal ponds could interact with epigenetics during development until metamorphosis. Once adult, salamanders live in the forest soil and are then exposed to different ecological and physicochemical factors. In addition, the sampled individuals were adults that may have developed over different breeding seasons since salamanders are iteroparous.

To circumvent this, the sampling site was considered as a proxy for environmental conditions, and all pairwise comparisons between sites are different from the same extent. This appears as a reasonable compromise given the distribution of sites of a given lineage according to the different sets of environmental factors: The sites dominated by the same lineage are globally very different from each other. However, it can be noted that the sites dominated by lineage C (M04 and M05) are more similar, whereas those dominated by lineage D (E01, E02, and E03) are more different.

### Genetic Variation

A second crucial factor to consider is the genetic differentiation of populations, which must be comparable between GE+ and GE− lineages. Both sampling sites hosting individuals from lineages B and E are genetically isolated from each other since sites M01 and M02 are located on different islands. There is a large geographic distance between sites hosting lineage D (E01, E02, and E03), while the landscape between sites hosting lineage A is highly urbanized. Only the sites hosting lineage C are not completely isolated from each other, but M04 and M05 are still 3.6 km apart and separated by a road and grasslands, which are typically avoided by *Ambystoma* salamanders [39]. Salamanders from the genus *Ambystoma* display strong fidelity to their breeding sites [40], and the dispersing individuals move on average only 150 m from their breeding ponds during breeding season, and rarely over 300 m [32, 39–42].

Depending on whether they perform genome exchange or not, lineages are characterized by different genetic diversity among sites according to microsatellite markers [27]. Clonal lineages A and D-2n are characterized by a very low diversity within the populations (one main genotype and a few alternative genotypes with low frequency). Lineages performing genome exchange are highly diversified, and most of the individuals are genetically different one from each other. However, individuals of the GE− lineages differ by a few stepwise mutations at multilocus genotypes, while most of the genotypes of the GE+ lineages differ by the male genome incorporated.

### Epigenetic Variation

The results revealed a marked difference in epigenetic variation between lineages with and without genome exchange. When genetics and ploidy level are taken into account, sample sites have a significant effect only on the environmentally induced epigenetic variation of clonal lineages A and D-2n. The three lineages that performed genome exchange (B, C, and E) did not display significant epigenetic differences among sites once the effects of genetics and ploidy were removed.

Individuals of lineage A are triploids; their genome includes a haplome from a paternal leakage in addition to the hybrid genome. It appears difficult to determine when the genome from paternal leakage was incorporated in this lineage. However, highly divergent alleles from the main microsatellite allele’s distribution of unisexuals were found in the sexual sympatric individuals [27]. Those highly divergent alleles were found in some unisexuals of the GE+ lineages, but were absent in lineage A. Sympatric unisexual and sexual individuals are then genetically distinct. In addition, individuals of this lineage harbor the same multilocus genotype across sites, indicating that paternal leakage predated colonization of the sites. We can therefore presume that the additional haplome of this lineage is not locally adapted to these sites, or at least some of them, since the sites where lineage A was found have different ecological conditions (Fig. 2).

It is however more straightforward with lineage D-2n. Despite their relative remoteness from the other unisexual populations, individuals of lineage D-2n harbor a hybrid genome genetically equidistant from other lineages. Since genome exchange events did not occur locally [27], the hybrid genome of these lineages is not expected to be locally adapted to their current and local environmental conditions. Nevertheless, individuals of lineage D-2n were detected in geographically distant and ecologically different sites. They also harbor significant epigenetic difference among sites, indicating that they strongly relied on environmentally induced epigenetic variation in the absence of a locally adapted haplome.

Individuals of the other lineages are likely not impervious to environmentally induced epigenetic variation, but they relied less on plasticity than clonal individuals. Lineages performing genome exchange can create genetic variation by replacing one of their haplomes with the haplome from a sexual species. This process has numerous advantages: It can purge deleterious alleles and increase genetic variation at the population scale. However, an important benefit is to gain a locally adapted haplome.

### Acclimatization vs. Adaptation

Several authors have argued that no relationship exists between phenotypic plasticity and genetic diversity since they are both traits that act independently to cope with different levels of environmental changes [17, 19, 43–45]. However, this hypothesis could not be applied to organisms without genetic variation. Macdonald and Chinnappa [15] argued that the survival of organisms with very low genetic variation relies on their high capacity for plasticity. Since such organisms rely only on other mechanisms than genetic diversity to generate phenotypic variation, selection would have favored lineages with a strong potential for alternative sources of phenotypic variation.

Kleptogenetic lineages, as partially sexual organisms, can use either genetic or epigenetic variation, as well as their potential interactions. So, why do GE+ lineages put plasticity apart? One possible answer could be the cost of plasticity. Plasticity is expected to increase fitness by producing alternative phenotypes according to environmental conditions. However, this strategy is profitable only when conditions are fluctuating and when the changes are predictable [9, 46]. Moreover, the potential for plasticity itself is expected to reduce fitness; theoretical models and empirical studies suggest a cost of the potential for plasticity in terms of reduced growth and increased developmental instability [47–50].

## Conclusion

Lineages with and without genome exchange provide a useful model to assess the role of genetic variation in plasticity. Clonal organisms strongly rely on plasticity to adjust their phenotypes to a given environment. On the contrary, organisms that have the opportunity to use locally adapted alleles to generate their phenotypes depend less on plasticity. The importance of plasticity in an organism’s strategy seems to depend not only on the amount of genetic diversity but also on the adequacy of alleles for the local environment.

## Supplementary Material

Supplementary Data
