# Supplementary material for: Effect of a locally adapted genome on environmentally induced epigenetic variation
Source: Environ Epigenet. 2018 Nov 26;4(4):dvy025. doi: 10.1093/eep/dvy025 (PMC6255975; doi:10.1093/eep/dvy025)
Supplement: Supplementary Data [file dvy025_supp.zip › dvy025_Supp_Additionnal_file_1.docx]

Table S1: Number of unisexuals of the subsample from each sites. The letters refer to the lineages described in Beauregard & Angers 2018. The numbers in parenthesis refer to the number of each individuals according to their ploidy levels: diploids, triploids and tetraploids (2n, 3n, 4n).

| **Sites** | **Lineages** | | | | | **Total** |
| --- | --- | --- | --- | --- | --- | --- |
|  | **A** | **B** | **C** | **D** | **E** |  |
| **M01** |  | 11 (3,8,0) |  |  | 6 (2,3,1) | 17 |
| **M02** | 4 (0,4,0) | 2 (0,1,1) |  |  | 20 (5,13,2) | 26 |
| **M03** |  |  |  |  | 2 (0,2,0) | 2 |
| **M04** |  |  | 16 (0,15,1) |  |  | 16 |
| **M05** |  |  | 17 (0,16,1) |  |  | 17 |
| **M06** | 11 (0,10,1) |  |  |  |  | 11 |
| **M07** | 15 (0,15,0) |  |  |  |  | 15 |
| **E01** |  |  |  | 6 (6,0,0) |  | 6 |
| **E02** |  |  |  | 4 (4,0,0) |  | 4 |
| **E03** |  |  |  | 11 (0,11,0) |  | 11 |
| **Total** | **30 (0,29,1)** | **13 (3,9,1)** | **33 (0,31,2)** | **21 (10,11,0)** | **28 (7,18,3)** | **125** |

Table S2: Details on environmental variables collected. Environmental variables were collected from May to August 2014. Chemical aquatic samples were taken with the YSI 556-MPS probe. Terrestrial variables were taken on two 20 x 20 m quadrats, located 50 m from the water body and 50 m apart.

| **Variable** | **Units** | **Description** | |
| --- | --- | --- | --- |
|  |  |  |  |
| Mean_conductivity | mS/cm | Conductivity measured with Probe YSI 556-MPS, average of the four months |  |
| Mean_O2 | mg/L | Dioxygen concentration measured with Probe YSI 556-MPS, average of the four months |  |
| Mean_water_pH | (pH) | Water pH measured with Probe YSI 556-MPS, average of the four months |  |
| Mean_ORP | mV | Oxydoreduction potential measured with Probe YSI 556-MPS, average of the four months |  |
| Pond_substratum.humus/clay Soil_substratum.humus/clay |  | Type of pond or terrestrial substrate : qualitative variable: 1 = humus ; 0 = clay |  |
| Water_level_June Water_level_July | cm | Change of water level from a reference point (May = 0): values in June and July |  |
| Water_presence_July |  | Presence/absence of water in July: qualitative variable: 1 = more than 15 cm at deepest point; 0.5 = less than 15 cm ; 0 = the pond is dry |  |
| Water_connectivity |  | Connexion with other water sources: qualitative variable: 1 = the pond is linked to a stream ; 0 = the pond is isolated |  |
| Herbaceous_presence Tree_presence |  | Presence of herbaceous plants or trees in the pond: qualitative variable: 1 = presence; 0 = absence |  |
| Forest_cover | % | Canopy cover of the pond : semiquantitative variable: 0%, 25%, 50%, 75%, 100% |  |
| Mean_soil_pH | (pH) | Average of the five data records for each quadrat for the four months |  |
| Drainage |  | Drainage rating of the quadrates : semiquantitative variable: according to Emberger et al. 1968 |  |
| %_conifers | % | Number of conifers/Number of trees in the quadrats * 100 |  |
| Mean_3m_cover Mean_1m_cover | % | Vegetation cover at >3m (3m) and between 0 to 2m (1m): semiquantitative variable: 0% to 100%, classes of 10% |  |
| Mean_hour | h | To account for the time of the day: number of hour since midnight |  |

Emberger L, Godron M, Daget P (1968). Code pour le relevé méthodique de la végétation et du milieu: Principes et transcription sur cartes perforées: Éditions du Centre national de la recherche scientifique.

Table S3: Environmental and genetic effect on the epigenetic pattern of unisexual individuals. Effects for each lineage determined are listed.

| **Lineage** | **Environmental effect** | **Ajusted R-square** | **p-value** | **Genetic effect** | **Ajusted R-square** | **p-value** |
| --- | --- | --- | --- | --- | --- | --- |
|  |  |  |  |  |  |  |
| **A** | pure | 0.06328 | 0.018 * | pure | 0.00000 | NA |
|  | global | 0.06555 | 0.008 ** | global | -0.00192 | 0.422 |
|  |  |  |  |  |  |  |
| **B** | pure | 0.00000 | NA | pure | 0.20549 | 0.156 |
|  | global | 0.02922 | 0.184 | global | 0.17976 | 0.152 |
|  |  |  |  |  |  |  |
| **C** | pure | 0.00220 | 0.367 | pure | 0.23286 | 0.001 *** |
|  | global | 0.03991 | 0.016 * | global | 0.24028 | 0.001 *** |
|  |  |  |  |  |  |  |
| **D** | pure | 0.09327 | 0.001 *** | pure | 0.01118 | 0.416 |
|  | global | 0.21813 | 0.001 *** | global | 0.08059 | 0.072 |
|  |  |  |  |  |  |  |
| **E** | pure | -0.05478 | 0.894 | pure | 0.01441 | 0.422 |
|  | global | 0.02901 | 0.151 | global | 0.12318 | 0.081 |
